# Dietary marine-derived long-chain monounsaturated fatty acids and cardiovascular disease risk: a mini review

**DOI:** 10.1186/s12944-016-0366-5

**Published:** 2016-11-22

**Authors:** Zhi-Hong Yang, Beatrice Emma-Okon, Alan T. Remaley

**Affiliations:** 1 Lipoprotein Metabolism Section, Cardio-Pulmonary Branch, National Heart, Lung and Blood Institute (NHLBI), National Institutes of Health (NIH), Bethesda, MD 20892-1666 USA; 2 Central Research Laboratory, Tokyo Innovation Center, Nippon Suisan Kaisha, 32-3 Nanakuni 1 Chome Hachioji, Tokyo, 192-0991 Japan

**Keywords:** Long-chain monounsaturated fatty acids, Cardiovascular disease, Atherosclerosis, Peroxisome proliferator-activated receptor signaling pathway, Inflammation

## Abstract

Regular fish/fish oil consumption is widely recommended for protection against cardiovascular diseases (CVD). Fish and other marine life are rich sources of the cardioprotective long-chain n-3 polyunsaturated fatty acids (n-3 PUFA) eicosapentaenoic acid (C20:5 n-3; EPA) and docosahexaenoic acid (C22:6 n-3; DHA). The lipid content and fatty acid profile of fish, however, vary greatly among different fish species. In addition to n-3 PUFA, certain fish, such as saury, pollock, and herring, also contain high levels of long-chain monounsaturated fatty acids (LCMUFA), with aliphatic tails longer than 18 C atoms (i.e., C20:1 and C22:1 isomers). Compared with well-studied n-3 PUFA, limited information, however, is available on the health benefits of marine-derived LCMUFA, particularly in regard to CVD. Our objective in this review is to summarize the current knowledge and provide perspective on the potential therapeutic value of dietary LCMUFA-rich marine oil for improving CVD risk factors. We will also review the possible mechanisms of LCMUFA action on target tissues. Finally, we describe the epidemiologic data and small-scaled clinical studies that have been done on marine oils enriched in LCMUFA. Although there are still many unanswered questions about LCMUFA, this appears to be promising new area of research that may lead to new insights into the health benefits of a different component of fish oils besides n-3 PUFA.

## Background

Cardiovascular disease (CVD) is the leading global cause of death, accounting for 17.3 million deaths per year, and is expected to grow to more than 23.6 million by 2030 [1]. According to a recent American Heart Association analysis, 40.5% of the US population is projected to develop some form of CVD by 2030. Indirect costs from CVD are projected to increase by 61%, from $172 billion in 2010 to over $276 billion in 2030 [2]. In addition, both metabolic syndrome and type 2 diabetes confer an increased risk of coronary heart disease and CVD [3, 4]. Thus, the mortality associated with CVD and its rising costs illustrate the importance of CVD prevention and the necessity for identifying effective ways to prevent its occurrence.

Fish and fish oil consumption has been shown in multiple studies to be related to reduced incidence of sudden cardiac death and total mortality [5–7]. Epidemiologic studies have also found that a high intake of small and medium size fish (mackerel, sardine, saury and eel) was associated with a lower risk of type 2 diabetes [8]. Currently, the American Heart Association recommends eating fatty fish, such as salmon, mackerel, and herring, at least twice a week to promote heart health [9]. Fish and fish oils are known to be enriched in long-chain n-3 polyunsaturated fatty acid (PUFA), such as eicosapentaenoic acid (C20:5 n-3; EPA) and docosahexaenoic acid (C22:6 n-3; DHA). The therapeutic use of n-3 PUFA has been extensively studied in a wide variety of disease conditions, including CVD. Potential mechanisms whereby n-3 PUFA may reduce risk for CVD include their beneficial effects on lipid and lipoprotein metabolism, blood pressure, platelet function, arterial cholesterol delivery, vascular function, and inflammatory responses [10, 11]. Fish lipids, however, also contain varying amounts of other unusual types of fatty acids (FA) that are not commonly found in other food sources [12]. For example, saury [13], pollock [14], herring [15], capelin [16], and sprats [17], as well as marine mammals, such as seals and whales [18], are all enriched in long-chain monounsaturated fatty acids (LCMUFA) that originate from their food source, such as zooplankton [19, 20] (Table 1). LCMUFA are defined as monounsaturated fatty acid isomers with aliphatic tails at least 20 carbons (Table 2), with n-11 LCMUFA as the most abundant component. Although the n-9 LCMUFA isomers, gondoic acid (C20:1 n-9) and erucic acid and (C22:1 n-9), are also found in vegetable oils from mustard seed and rapeseed, the major LCMUFA enriched in marine sources are the n-11 series, gadoleic acid (C20:1 n-11) and cetoleic acid (C22:1 n-11) [21].

**Table 1:** Fatty acid profile of marine sources enriched in LCMUFA

FA (%)	Calanus Oil	Seal Oil	Whale Oil	Herring oil	Mackerel oil	Saury oil	Pollock oil
Reference	[19]	[59]	[59]	[15]	[30]	[13]	[14]
C14:0	9.1	6.5	5.3	9.8	8.0	N.S.	N.S.
C16:0	7.4	10.1	7.9	14.3	13.9	9.2	9.8
C18:0	N.S.	1.4	1.8	1.9	2.6	1.7	1.7
Σ SFA	18.2	18	15.3	26.1	29.8	10.9	11.5
C16:1	16.2	16.8	8.8	5.0	4.2	2.9	6.1
C18:1	3.3	22.9	16.3	8.4	14.9	5.8	14.3
C20:1 n-11	N.S.	N.S.	1.7	12.2^b^	12.2	12.1	9.1
C20:1 n-9	12.2	10.9	15.3		0.8	3.1	3.3
C22:1 n-11	12.5	N.S.	9.8	20.7^c^	18.1	18.5	12.3
C22:1 n-9	2.5	N.S.	0.9			1.0	1.6
Σ LCMUFA^a^	27.2	10.9	27.7	32.9	31.1	34.7	26.3
Σ MUFA	58.9	50.6	54.8	46.4	50.2	43.4	46.7
C18:2 n-6	0.6	1.8	1.4	1.7	1.8	1.6	1.3
C20:4 n-6	N.S.	0.5	0.3	N.S.	N.S.	0.6	N.D.
Σ n-6 PUFA	0.6	2.3	2	1.7	1.8	2.2	1.3
C18:3 n-3	0.1	0.6	1.2	1.1	1.5	1.2	1.1
C20:5 n-3	8.6	9	4.3	6.3	5.4	1.6	1.2
C22:5 n-3	N.S.	5.2	2.3	N.S.	0.4	6.1	10.3
C22:6 n-3	9.3	11.7	7.9	6.8	7.8	11.8	7.9
Σ n-3 PUFA	19.5	26.5	18.7	17.3	16.5	20.7	20.5

^a^LCMUFA is shown as the sum of C20:1 and C22:1 isomers; ^b^presented as C20:1; ^c^presented as C22:1. *LCMUFA* long-chain monounsaturated fatty acid, *MUFA* monounsaturated fatty acid, *N.D.* not detected, *N.S.* not shown, *PUFA* polyunsaturated fatty acid, *SAF* saturated fatty acid

**Table 2:** List of most common LCMUFA isomers found in marine source

Common name	Lipid name	Systematic name
Gadoleic acid	C20:1 n-11	cis-9-icosenoic acid
Cetoleic acid	C22:1 n-11	cis-11-docosenoic acid
Gondoic acid	C20:1 n-9	cis-11-eicosaenoic acid
Erucic acid	C22:1 n-9	cis-13-docosenoic acid

Saury is one of the most highly enriched fish in LCMUFA and is widely consumed in Asia [22]. Herring, which is among the main commercial fish species in European countries, and Alaska pollock, which is the primary target for the fish industry in the United States, are also enriched in LCMUFA [23, 24]. In addition, some of the more widely consumed fish species, such as salmon and cod, also contain a significant amount of LCMUFA [14, 25]. Thus, a considerable amount of LCMUFA is consumed from various fish sources. Although numerous reports and reviews in recent years have showed beneficial effects of marine-derived n-3 PUFA on CVD and many other chronic diseases, only a limited number of studies have focused on the health impact of the consumption of LCMUFA. The purpose of this review is, therefore, to describe the experimental and clinical evidence on the effect of LCMUFA-rich diet on human health.

## Incorporation of LCMUFA into plasma and organ lipids

Compared with n-11 LCMUFA (C20:1 n-11 and C22:1 n-11), which can only be derived from the diet, n-9 LCMUFA (C20:1 n-9 and C22:1 n-9) can also be formed by *de novo* synthesis, by the action of FA elongases on oleic acid (C18:1 n-9) [26]. Although earlier animal studies showed that diets enriched in the LCMUFA isomer C22:1 caused a transient lipidosis in some organs, lipidosis disappeared upon continued feeding, possibly due to increased activity of peroxisomal β-oxidation [27]. A recent animal feeding study from our group showed that the LCMUFA-rich diet resulted in a small but significant increase in each LCMUFA isomer in plasma and vital organs, such as liver, skeleton muscle, and duodenum, with the most prominent changes occurring in adipose tissues [28]. Similarly, generally MUFA is also enriched in adipose tissue [29], because of either its greater entrance into adipocytes or because of a putative desaturation process of saturated FA by the steraoyl desaturase (SCD1). Compared with organ levels of LCMUFA, less LCMUFA are found in plasma, suggesting a possible rapid metabolism of these monoenoic acids. This hypothesis is supported by human studies. An early study conducted by von Lossonczy et al. [30] showed that the plasma LCMUFA was not detected in serum lipid fractions, such as TG and sterol esters, in healthy subjects fed mackerel diet for 3 weeks, despite of high content of LCMUFA (31% (*w/w*)) in the mackerel fat. In another study, plasma chylomicron cetoleic acid (C22:1 n-11) levels peaked at hour 3–6 (10.6% ± 4.5), and dropped to near baseline (0.03% ± 0.08) at 12–24 h (0.13% + 0.05) after fasting subjects consumed 50 ml of herring oil enrich in LCMUFA (22.3% cetoleic acid) [31]. We also found that a single serving of LCMUFA-rich saury diet (11 g of LCMUFA and 5.6 g of n-3 PUFA in the saury meal) resulted in a rapid 13-fold increase in plasma LCMUFA levels 2 h after the meal and then declined sharply [32]. The postprandial plasma levels of LCMUFA dropped significantly at 24 h, although it was still significantly higher than baseline (0.37% ± 0.02 at 24 h vs. 0.17% ± 0.05 at baseline, *p* < 0.01). In contrast, plasma n-3 PUFA levels peaked at 6 h and increased only by 1.1-fold after saury ingestion and then gradually declined. At 24 h post-ingestion, plasma n-3 PUFA levels rose by 67% compared with the pre-ingestion values (10% ± 0.6 at 24 h vs. 6% ± 0.7 at baseline, *p* < 0.01). Overall, these results suggest that different metabolic pathways may be responsible for the mobilization rates of different fish oil-derived fatty acids. In general, it appears that LCMUFA are rapidly catabolized and thus do not accumulate in plasma. Braekkan et al. also reported that no direct relation was found between the dietary fat types and fatty acid deposition [33]. In contrast to fatty acids with shorter chain length and higher unsaturation that seemed to increase plasma deposition, LCMUFA had decreased plasma deposition probably due to rapid metabolism through retroconversion and or chain elongation [27].

## Animal studies

### LCMUFA-rich marine oil diet and CVD risk factors

**Fig. 1:**
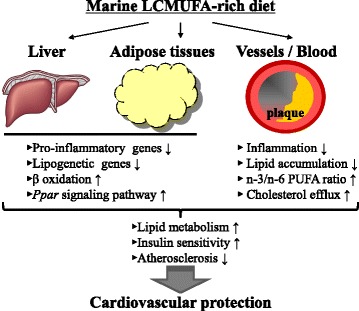
Beneficial effects of marine LCMUFA-rich diet. LCMUFA suppressed lipogenesis and inflammation, and promoted fatty acid oxidation PPAR signaling pathway at gene expression level in liver and white adipose tissues. In the vessels, LCMUFA suppressed lipid deposition and macrophage accumulation. LCMUFA also improved plasma lipid and cytokine profiles, as well as n-3/n-6 PUFA ratio. All these mechanisms accounted for the LCMUFA-mediated improvement in lipid metabolism, insulin sensitivity, and atherosclerosis

Several animal studies have reported that LCMUFA–rich marine oil, improved CVD risk factors. A summary of these findings and the possible mechanism of action of LCMUFA on target tissues is shown in Fig. 1. For example, a high-fat fed C57BL/6J mice treated with saury oil for either short term (6-weeks) or long term (18-weeks) showed major improvements in several features related to metabolic syndrome [28, 34]. A 10% (*w/w*) of supplementation of saury oil (equivalent to appropriate 3.5% (*w/w*) LCMUFA) in a high-fat diet ameliorated diet-induced hyperinsulinemia and dyslipidemia compared to high-fat control diet. Saury oil diet resulted in a significant increase in LMCUFA levels, especially n-11 LCMUFA, in plasma and in organs (liver, adipose tissues and skeleton muscle). Suppression of genes related to adipogenesis and induction of genes involved in fatty acid oxidation and insulin signaling with saury oil supplementation were also associated with improvements in glucose and lipid metabolism. Similarly, 6-week treatment of 15% (*w/w*) of LCMUFA-rich pollock oil (equivalent to appropriate 3.9% (*w/w*) LCMUFA) in diet-induced obese mice increased organ levels of LCMUFA, and suppressed the rise in proatherogenic LDL-cholesterol without decreasing anti-atherogenic levels of HDL-cholesterol [35]. An attenuation in hepatic steatosis and a down-regulation of hepatic genes involved in cholesterol and lipid synthesis by the pollock oil diet most likely contributed to these findings. In addition, Gabrielsson et al. fed LDLR-deficient mice herring fillet or beef for 16 weeks, and investigated the effect of dietary herring on plasma lipid levels and atherosclerosis [36]. The major differences in fatty acid composition between herring and beef diet were the enrichment of long-chain n-3 PUFA (herring diet: 4.9% EPA and DHA vs. beef diet: Not Detected) and LCMUFA (herring diet: 3.4% C20:1 and C22:1 vs. beef diet: Not Detected) in herring diet. They showed that herring diet compared to the beef diet led to lower plasma triglyceride (TG) and Very-low-density lipoprotein (VLDL)-cholesterol levels and higher plasma High-density lipoprotein (HDL)-cholesterol levels, along with less atherosclerotic lesions. A recent study by Eilertsen et al. used marine mammal oil, and the results showed that atherogenesis was inhibited in apoE-deficient mice fed diet supplemented with 1% of seal oil combined with extra virgin oil (EVO/n-3), compared with diet supplemented with 1% corn oil or without any supplement (control). Besides long-chain omega-3 PUFA, such as EPA and DHA, the EVO/n-3 oil was also enriched in LCMUFA (C20:1 in EVO/n-3 diet was 2-fold higher than that in control or corn oil-rich diet), suggesting that in addition to n-3 PUFA that the LCMUFA in the seal oil may also contribute to the protection against atherosclerosis [37]. In addition to the studies using LCMUFA-rich fish oils or marine mammal oils, some studies also focused on the health impact of zooplankton-derived oils. *Calanus finmarchicus* is the most abundant herbivorous zooplankton that that are enriched in both n-3 PUFA and LCMUFA [38]. Several studies showed beneficial effect of dietary Calanus oil in CVD risk, such as reducing atherosclerotic plaque formation, abdominal fat accumulation and hepatic steatosis, and improving glucose tolerance in mice through multiple mechanisms, including regulation of inflammatory response-associated gene expression in livers and adipose tissues [39–41]. Nevertheless, because these marine oils also contain considerable amounts of n-3 PUFA and intake of these marine oils increased plasma and organ levels of EPA and DHA, one cannot exclude the possibility that the benefit from this diet was only due to n-3 PUFA consumption. Further animal studies using purified LCMUFA are necessary to better understand the functional relationships between dietary LCMUFA and CVD risk factors.

### Dietary LCMUFA concentrate oil and CVD risk factors

Only a few studies have been conducted to investigate the impact of dietary marine-derived LCMUFA on metabolic disorders (Table 3). Our group concentrated LCMUFA (LCMUFA: 60~70%; total n-3 PUFA: <1%) from saury oil and estimated its effect in animal models on various metabolic and inflammatory parameters, as well as atherosclerosis. A 5% (*w/w*) LCMUFA supplemented western diet for 6-weeks in diet-induced obese mice improved insulin resistance and reduced blood lipids compared to western diet control. These changes were attributed to favorable alterations in the mRNA level of genes related to glucose/lipid metabolism and inflammation [42]. Similarly, chow diet supplemented with 4% (*w/w*) LCMUFA concentrate for 8-weeks in type II diabetic mice alleviated several metabolic parameters compared to soybean oil diet [43]. Along with reduced hyperinsulinemia, hyperleptinemia, and adipocyte size, the LCMUFA diet down-regulated inflammatory genes, and up-regulated *Pparg* and its target genes. *Pparg*, an isoform in the PPAR family, is known to play a crucial role in regulating adipocyte differentiation and fatty acid metabolism [44, 45]. The LCMUFA-induced improvements in diabetes may, therefore, be possibly related to the upregulation of *Pparg* and its target genes. Upregulation of PPAR family genes by dietary LCMUFA was also observed in ApoE-deficient mice and LDLR-deficient mice [46]. Both a higher dose 5% (*w/w*) or a lower dose 2% (*w/w*) LCMUFA supplementation in western diet significantly suppressed atherosclerotic development and upregulated key gene expression in PPAR signaling pathway, such as *Pparα* and *Pparγ*. PPARs are known to protect against atherosclerosis partly by regulating inflammation and enhancing ABCA1-dependent cholesterol efflux from macrophages in the arterial wall [47]. Our data showed that LCMUFA diet decreased plasma inflammatory cytokine levels, and enhanced cholesterol efflux of apoB-depleted plasma in LDLR-deficient mice fed a LCMUFA diet by the ABCA1 transporter. It is important to note that when 2% of olive oil enriched in shorter-chain MUFA oleic acid was supplemented in western diet, no significant difference was found in atherosclerosis between the olive oil group and western diet fed control group of LDLR-deficient mice, suggesting that the effect of MUFA on CVD risk may differ by carbon chain lengths. Further studies are needed to elucidate the potential regulatory effects of individual LCMUFA isomers on modulating PPAR levels.

**Table 3:** Summary of animal studies using LCMUFA concentrate oil (LCMUFA: 58~80%; n-3 PUFA: <0.1%)

Ref.	Animal model	Diet	Term	Health outcomes of LCMUFA diet (*p* < 0.05 vs. control)
[42]	Diet-induced obese mice C57BL/6J (*n* = 10/group)	Hight-fat diet (42% energy from fat) supplemented 5% LCMUFA or not (control)	6 weeks	Plasma TC, LDL-C reduction; Insulin resistance improvement; Anti-inflammation (Decreased plasma resistin levels and inflammatory gene expressions); Increase in n-3 PUFA and decrease in n-6 PUFA in liver
[43]	Spontaneous Type II diabetic mice KKAy (*n* = 10/group)	Chow diet supplemented 4% LCMUFA or soybean oil (control)	8 weeks	Plasma TC, FFA, insulin and leptin reduction; Adipocyte size reduction; Upregulation of *Pparg* family genes
[46]	ApoE-KO mice (*n* = 13-15/group)	Western diet (42% energy from fat) supplemented 5% LCMUFA or not (control)	12 weeks	Plasma TC, LDL-C reduction; Atherosclerotic lesion area reduction; Anti-inflammation (Decreased macrophage and inflammatory gene expression in aorta); Upregulation of PPAR signaling pathway in liver
[46]	LDLR-KO mice (*n* = 12/group)	Western diet (42% energy from fat) supplemented 2% LCMUFA, olive oil, or not (control)	12 weeks	No change in plasma lipid levels; Atherosclerotic lesion area reduction; Anti-inflammation (Decreased plasma inflammatory cytokine levels and in aorta); Upregulation of PPAR signaling pathway in liver; Increase in n-3 PUFA and decrease in arachidonic acid (C20:4 n-6) in plasma and liver
[48]	Diet-induced obese Wistar rats (*n* = 6/group)	High-fat diet (42% energy from fat) supplemented 6.5% LCMUFA, n-3 PUFA, or not (control)	3 weeks	No change in plasma lipid levels; Stimulation of peroxisomal β-oxidation in liver; Increase in n-3 PUFA and decrease in arachidonic acid (C20:4 n-6) in plasma and liver

*FFA* free fatty acids, *KO* knockout, *LCMUFA* long-chain monounsaturated fatty acids, *LDL-C* LDL cholesterol, *PPAR* peroxisome proliferator-activated receptor, *PUFA* polyunsaturated fatty acids, *TC* total cholesterol

Interestingly, intake of LCMUFA concentrate has been shown to increase plasma/hepatic EPA and DHA levels and decrease arachidonic acid (C20:4 n-6) levels, although there were only trace levels of long-chain n-3 PUFA in the LCMUFA concentrate oil (EPA and DHA <0.5%) [42, 43, 46]. Mobilization of PUFA after LCMUFA diet feeding was also observed by Halvorsen et al. [48]. In this study, a 3-week feeding of LCMUFA concentrate oil to rats resulted in a doubling of the EPA/arachidonic acid ratio in liver and plasma phospholipids compared to lard diet. Arachidonic acid, one of the major components of n-6 PUFA that competes with n-3 PUFA for several physiological processes, is reported to increase inflammatory signals possibly through one of its myriad metabolites and has been associated with metabolic/cardiovascular disorders [49]. A low n-3/n-6 PUFA ratio has also been shown to promote the pathogenesis of many diseases, including cardiovascular disease and inflammatory diseases [50]. Thus, LCMUFA-rich marine oils may also be beneficial in regard to CVD by their effect on circulating and organ levels of PUFA.

### Comparison of the effect of dietary n-3 PUFA-rich and LCMUFA-rich marine oils on CVD risk factors

Both long-chain n-3 PUFA and LCMUFA are the main fatty acid fractions in some fish species and marine mammals, and the individual actions of n-3 PUFA and LCMFUA need to be better understood. To date, only a few studies have compared the effect of marine-derived n-3 PUFA and LCMUFA on CVD risk factors in animal models. In two earlier studies [48, 51], rats were fed lard diet supplemented with 6.5% of concentrated fish oil-derived n-3 PUFA (85% EPA and DHA), LCMUFA (80% C20:1 and C22:1), or not (control) for 3-weeks. The peroxisomal β-oxidation in livers from mice in both n-3 PUFA and LCMUFA diet groups were significantly enhanced compared with that of the lard diet group, with the n-3 PUFA diet group showing a greater increase. Previous studies also showed stimulatory effect of LCMUFA-rich oils on peroxisomal β-oxidation in the heart [52]. FA are known to be degraded by both mitochondrial (primarily FAs with carbon chain length shorter than C20) and peroxisomal β-oxidation (primarily FAs with carbon chain length greater than C20), and these two pathways are partly controlled at the gene regulatory level [53, 54]. The nuclear receptor PPARα has been reported to have an important role in the transcriptional control of genes involved in cardiac FA oxidation, and long-chain FA, such as EPA and DHA and their derivatives, serve as PPARα ligands [55, 56]. The upregulation of *Pparα* by dietary n-3 PUFA or LCMUFA, therefore, may account for the stimulation of peroxisomal FA oxidation. In another study investigating metabolism differences between dietary fish oil and seal oil, plasma and hepatic lipids and lipid peroxidation levels were markedly lower in hamsters fed seal oil-rich diet for 4-weeks compared to those fed fish oil [57]. One of the distinct differences between fish oil and seal oil was the fatty acid composition. Seal oil contains much higher levels of MUFA compared to fish oil (50.6% of MUFA in seal oil vs. 22.2% in fish oil). Because a considerable amount of shorter-chain MUFA (C18:1 n-9 and C16:1 n-7) were also contained in the MUFA fraction of seal oil besides LCMUFA (C20:1 n-9), it will be important to delineate the individual effects of each FA on the regulation of lipid metabolism and oxidative stress.

We recently analyzed the plasma lipid change in mice fed a lard diet supplemented with n-3 PUFA (97% EPA), LCMUFA (57% C20:1 and C22:1), or not (control) for 8-weeks [28]. Mice in both n-3 PUFA and LCMUFA diet groups showed significantly lower total cholesterol in plasma than mice in the lard diet group, and this effect was stronger in the n-3 PUFA diet group compared with the LCMUFA diet group. In contrast to EPA, which decreased plasma total cholesterol with a concomitant decrease in HDL-cholesterol, LCMUFA-rich diet did not decrease HDL-cholesterol. Although the lipoprotein response to LCMUFA has not been well investigated, several studies have shown that MUFA decrease plasma LDL-cholesterol without lowering HDL-cholesterol [58]. Nevertheless, the mechanism for the effect of LCMUFA-rich fish oil on HDL cholesterol and other lipoprotein changes is not fully understood and further studies are needed.

## Human studies

### Interventional studies

To date there are no published clinical studies that have directly evaluated the role of LCMUFA on improving CVD risk. A small number of studies have examined the plasma lipid and lipoprotein response to marine diets enriched in both n-3 PUFA and LCMUFA. Osterud et al. estimated the health impact of dietary marine oils (whale oil, seal oil and cod liver oil) contained in typical Eskimo diet in healthy subjects (*n* = 134), with whale oil mostly enriched in LCMUFA (28% LCMUFA vs. 18.7% n-3 PUFA) and cod liver oil mostly enriched in n-3 PUFA (28.7% n-3 PUFA vs. 18.2% LCMUFA) [59]. After 10-week intake of various oils (15 ml/day), whale oil consumption had the most profound effect on increasing plasma HDL-cholesterol compared to the baseline, along with a significant increase in plasma LCMUFA levels by 80% compared to control (no oil). In contrast, plasma TG decreased significantly only in cod liver oil group compared to the control, with most profound increased in plasma n-3 PUFA levels. In another study using marine mammal oil, healthy subjects (*n* = 19) consumed 20 g of vegetable oil (control) or seal oil enriched in omega-3 PUFA (26.2% (*w/w*) vs. 0.3% in control) and LCMUFA (15.2% (*w/w*) vs. 0.3% in control) for 42 days. Compared with control, the seal oil supplement group showed a modest beneficial effect on fibrinogen and protein C levels [60]. Similarly, a study by Bakken et al. showed that 2-week seal oil supplement (15 ml per day) attenuated microbubble-induced platelet aggregation compared with baseline in 11 healthy volunteers [61]. Besides marine mammal oils, several studies investigated the association between dietary fish oils enriched in n-3 PUFA and LCMUFA and CVD risk. A cross-over study by Childs and associates investigated the lipoprotein response to different types of fish oils (EPA-rich pollock oil and DHA-rich tuna and salmon blend oils) in healthy subjects [62]. Normolipidemic subjects consumed a diet enriched in butter fat, pollock oil, or either tuna or salmon-blend oil for 3-weeks, with a 4-week washout period between treatments. Interestingly, although both tuna and salmon blend oils used in the study were enriched in DHA (14.4% (*w/w*) in tuna oil, 9.6% in salmon blend oil, and 2.5% in butter), HDL responded differently in the tuna and salmon diet groups. Compared with butter diet, total HDL-cholesterol and HDL_2_-cholesterol decreased on the tuna diet, whereas there was no change in HDL-cholesterol and HDL_2_-cholesterol increased slightly in the salmon diet group. One factor that could possibly be responsible for this difference was the higher amount of LCMUFA in salmon blend oil (25% LCMUFA in salmon blend oil vs. 5.1% in tuna oil). The authors assumed that LCMUFA, as a MUFA family member, may act on plasma HDL similarly to oleic acid, which decreases LDL-cholesterol without decreasing HDL-cholesterol in human. Herring fish also contains a large amount of LCMUFA in its fillets, byproducts, or oil, and a few studies investigated the effect of herring diet on cardiovascular disease risk factors [63, 64]. The results showed that compared with the diet of matched pork and chicken diets, a 6-week herring-rich diet significantly raised HDL-cholesterol in healthy obese subjects. Because the study focused on the physiologic effect of n-3 PUFA contained in herring, no data were shown on the change of plasma LCMUFA levels after herring consumption, thus no definitive conclusion could be drawn on the possible correlation between plasma HDL-cholesterol and the LCMUFA consumption. Nevertheless, an important observation was that the content of MUFA levels was 2-fold higher in herring dishes (~15% of energy) compared to those in reference dishes (<5% of energy). Future studies on LCMUFA-rich fish/fish oil diet, particularly examining the difference in the change of lipid and lipoprotein between LCMUFA-rich fish/fish oil diet, are necessary to advance this field.

### Observational studies

The early epidemiologic studies starting in the 1960s, which first showed a lower incidence of coronary atherosclerosis and lower plasma lipid levels in certain ethnic groups, such as Greenland Eskimos, who ate a lot of fish [65, 66], also suggested a possible benefit from LCMUFA consumption. Analysis of fatty acid composition of the Eskimo food revealed that in addition to n-3 PUFA, dietary LCMUFA (C20:1 and C22:1) amount per day in a typical Eskimo food was significantly higher (13-fold) than that found in Danish control group [67]. Besides EPA and DHA, a significantly larger proportion of circulating LCMUFA (C20:1) was found in the serum of the Greenland Eskimo living in Greenland compared to another control group of Eskimos living in Denmark or a reference group of Caucasian Danes [68]. Recent studies on the association between red blood cell (RBC) fatty acid levels and incident coronary artery disease in the Physician’s Health Study also showed a strong inverse association between red blood cell LCMUFA consumption and coronary artery disease [69]. The RBC content of C22:1 n-9 showed significant inverse associations with coronary artery disease, even after being adjusted for RBC n-3 PUFA, saturated fatty acids, and de novo derived MUFA (C16:1 n-7 and C18:1 n-9). A similar inverse association was also observed between RBC C20:1 n-9 and coronary artery disease, although the significance disappeared after correction for multiple comparisons. In another cohort-based analysis of dietary FA intake and paraoxonase 1 (PON1), dietary gadoleic aicd (C20:1 n-11) intake was positively associated with PON1 activity in 1548 participants from Carotid Lesion epidemiology and Risk (CLEAR) study, whereas PUFA (EPA and arachidonic acid) were negatively associated with PON1 activity [70]. PON1 is believed to be a key cardioprotective factor on lipoproteins [71]. PON1 protects LDL and HDL against oxidation, and lower serum PON1 levels were associated with higher susceptibility of LDL oxidation and atherosclerosis risk [72]. MUFA are also positively associated with PON1 in mammals, and in vitro studies suggested that MUFA may bind to a protective site on PON1 and prevents its inactivation by oxidation [73].

Perreault et al. found that the relative percentage of C22:1 n-9 in serum were significantly lower in metabolically unhealthy obese participants compared to lean healthy and metabolically healthy obese participants in the Diabetes Risk Assessment study [74]. Metabolically healthy obese individuals had BMIs similar to metabolically unhealthy obese individuals, but they had lower risk for complications, such as inflammation, type 2 diabetes and CVD [75, 76]. The lipid parameters and circulating inflammatory markers in metabolically healthy obese individuals more closely resembled that of lean healthy individuals compared with metabolically unhealthy individuals, which may partly be attributed to the favorable change in FA profile. Two epidemiologic studies, however, showed that higher circulating erucic acid, but not gadoleic acid, were significantly correlated with higher incident congestive heart failure in the population from Cardiovascular Health Study (CHS) and the Atherosclerosis Risk in Communities Study, Minnesota subcohort (ARIC), respectively [77]. It is worth noting that the dominant constituents of marine-derived LCMUFA are n-11 LCMUFA, which are mainly found in marine source. The majority of LCMUFA that were examined in the observational studies were n-9 LCMUFA, which are generally found in both healthful and unhealthful food sources, such as fish, mustard, vegetable oils and processed meats [77]. Furthermore, in addition to LCMUFA, serum levels of some other fatty acids also changed in these observational studies, thus making it difficult to elucidate the exact relationship between LCMUFA intake and CVD risk. In summary, the current data from observational and interventional studies on LCMUFA intake are suggestive of a possible beneficial effect from LCMUFA on CVD health but at this point are inconclusive.

## Conclusion

Regular fish consumption is generally recommended to promote health, and fish oil is the most commonly used food supplement. Therefore, a more detailed understanding on the effect of the minor fatty acid components of fish oils, such as LCMUFA, on CVD risk factors is warranted. A limited number of in vivo animal studies have provided valuable evidence supporting the potential for LCMUFA-rich diet in the prevention of life-style related diseases, such as type 2 diabetes, metabolic syndrome, and atherosclerosis. A few human studies have also suggested a possible link between LCMUFA-rich diet and CVD risk protection, but further basic science and clinical research studies are needed to firmly establish the effect of LCMUFA-rich fish diet on atherosclerosis and CVD risk and its mechanisms of action.

## Abbreviations

CVD: Cardiovascular disease
DHA: Docosahexaenoic acid
EPA: Eicosapentaenoic acid
FA: Fatty acids
FC: Free cholesterol
KO: Knockout
LCMUFA: Long-chain monounsaturated fatty acids
MUFA: Monounsaturated fatty acids
PL: Phospholipid
PPAR: Peroxisome proliferator-activated receptor
PUFA: Polyunsaturated fatty acids
TC: Total cholesterol
TG: Triglyceride
